# 1,4-Bis(dimethyl­silyl)-2,5-diphenyl­benzene

**DOI:** 10.1107/S1600536810010913

**Published:** 2010-03-27

**Authors:** Lei Fang, Rui Wang, Li-Min Chen, Cai-Hong Xu, Shu-Hong Li

**Affiliations:** a Beijing National Laboratory for Molecular Sciences (BNLMS), Institute of Chemistry, Chinese Academy of Sciences, Beijing 100190, People’s Republic of China; b Graduate School of the Chinese Academy of Sciences, Beijing 100049, People’s Republic of China; c School of Chemical and Environmental Engineering, Beijing Technology and Business University, Beijing 100037, People’s Republic of China

## Abstract

The mol­ecule of the title compound, C_22_H_26_Si_2_, is centrosymmetric. The dihedral angle between the central benzene ring and its phenyl substituents is 67.7 (2)°. The crystal packing is stabilized by van der Waals forces.

## Related literature

For investigations on the effect of silyl substituents on the photophysics of *p*-terphenyls, see: Feng *et al.* (2007 ▶).
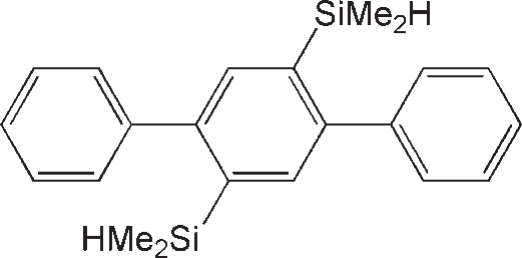


## Experimental

### 

#### Crystal data

C_22_H_26_Si_2_
*M* *_r_* = 346.61
Monoclinic, 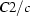

*a* = 14.8966 (3) Å
*b* = 6.0132 (1) Å
*c* = 26.1211 (6) Å
β = 123.166 (1)°
*V* = 1958.64 (7) Å^3^
*Z* = 4
Mo *K*α radiation
μ = 0.18 mm^−1^
*T* = 173 K
0.56 × 0.39 × 0.11 mm

#### Data collection

Rigaku R-AXIS RAPID IP area-detector diffractometer
Absorption correction: multi-scan (*ABSCOR*; Higashi, 1995 ▶) *T*_min_ = 0.905, *T*_max_ = 0.980
4032 measured reflections
2220 independent reflections
2009 reflections with *I* > 2σ(*I*)
*R*_int_ = 0.028

#### Refinement

*R*[*F* ^2^ > 2σ(*F* ^2^)] = 0.055
*wR*(*F* ^2^) = 0.118
*S* = 1.22
2220 reflections
111 parameters
H-atom parameters constrained
Δρ_max_ = 0.40 e Å^−3^
Δρ_min_ = −0.22 e Å^−3^

### 

Data collection: *RAPID-AUTO* (Rigaku, 2001 ▶); cell refinement: *RAPID-AUTO*; data reduction: *RAPID-AUTO*; program(s) used to solve structure: *SHELXS97* (Sheldrick, 2008 ▶); program(s) used to refine structure: *SHELXL97* (Sheldrick, 2008 ▶); molecular graphics: *SHELXTL* (Sheldrick, 2008 ▶); software used to prepare material for publication: *SHELXL97*.

## Supplementary Material

Crystal structure: contains datablocks global, I. DOI: 10.1107/S1600536810010913/gk2260sup1.cif

Structure factors: contains datablocks I. DOI: 10.1107/S1600536810010913/gk2260Isup2.hkl

Additional supplementary materials:  crystallographic information; 3D view; checkCIF report

## Figures and Tables

**Fig. 1.**
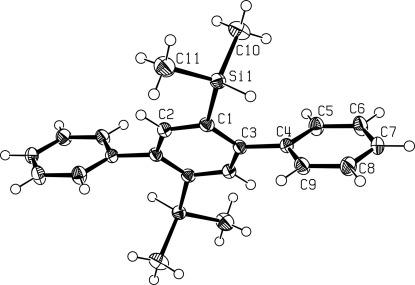
The structure of the title compound showing 50% probability displacement ellipsoids and the atom-numbering scheme. The unlabelled atoms can be generated by the symmetry operation -x, -y+2, -z.

## supplementary crystallographic information

### Comment

As part of our ongoing investigation on the effect of silyl substituents on the photophysics of *p*-terphenyls, we present the title compound bearing silyl substituents at the central phenyl ring (2,5-positions). Though analogues of the title compound were reported elsewhere (Feng *et al.*, 2007), their structures were not fully studied. The molecular structure of the title compound is shown in Fig.1. It is centrosymmetric, the centroid of the central benzene ring is located on an inversion center at 0,1,0. The dihedral angle between the benzene ring and phenyl substituents is 67.7 (2)°. The crystal packing is mainly stabilized by van der Waals forces.

### Experimental

A solution of 2,5-dibromo-1,4-diphenylbenzene (120 mg, 0.31 mmol) in anhydrous THF (10 ml) was added dropwise to a hexane solution of n-BuLi (2.5 M, 0.44 ml, 1.08 mmol) dropwise at -78 °C. The reaction mixture was stirred for 1 h and dimethylchlorosilane (118 mg, 1.24 mmol) was added via syringe at the same temperature and the mixture was allowed to warm to room temperature and stirred overnight. After being quenched with saturated NaHCO_3_ solution, the mixture was extracted with Et_2_O. The organic layer was washed with brine, dried over anhydrous MgSO_4_, filtered, and concentrated under reduced pressure. The mixture was passed through a silica gel column with hexane as an eluent, followed by further purification by recrystallization from ethanol to give 98 mg of the white product in 91% yield.

### Refinement

All H-atoms were located in electron-density difference maps. Carbon-bound H atoms were placed geometrically in idealized positions and refined using a riding model with C—H (methyl) 0.98, C—H (aromatic) 0.95 Å and with U_iso_(H) =1.2U_eq_(C). Located in the electron-density difference map H atom from the silyl group was refined using riding model with U_iso_(H) =1.2U_eq_(Si).

### Crystal data

**Table:**

C_22_H_26_Si_2_	*F*(000) = 744
*M**_r_* = 346.61	*D*_x_ = 1.175 Mg m^−^^3^
Monoclinic, *C*2/*c*	Mo *K*α radiation, λ = 0.71073 Å
Hall symbol: -C 2yc	Cell parameters from 4032 reflections
*a* = 14.8966 (3) Å	θ = 2.7–27.5°
*b* = 6.0132 (1) Å	µ = 0.18 mm^−^^1^
*c* = 26.1211 (6) Å	*T* = 173 K
β = 123.166 (1)°	Plate, colorless
*V* = 1958.64 (7) Å^3^	0.56 × 0.39 × 0.11 mm
*Z* = 4	

### Data collection

**Table:**

Rigaku R-AXIS RAPID IP area-detector diffractometer	2220 independent reflections
Radiation source: rotating anode	2009 reflections with *I* > 2σ(*I*)
graphite	*R*_int_ = 0.028
ω scans at fixed χ = 45°	θ_max_ = 27.5°, θ_min_ = 2.7°
Absorption correction: multi-scan (*ABSCOR*; Higashi, 1995)	*h* = −19→19
*T*_min_ = 0.905, *T*_max_ = 0.980	*k* = −7→7
4032 measured reflections	*l* = −33→33

### Refinement

**Table:**

Refinement on *F*^2^	Primary atom site location: structure-invariant direct methods
Least-squares matrix: full	Secondary atom site location: difference Fourier map
*R*[*F*^2^ > 2σ(*F*^2^)] = 0.055	Hydrogen site location: inferred from neighbouring sites
*wR*(*F*^2^) = 0.118	H-atom parameters constrained
*S* = 1.22	*w* = 1/[σ^2^(*F*_o_^2^) + (0.0432*P*)^2^ + 2.0232*P*] where *P* = (*F*_o_^2^ + 2*F*_c_^2^)/3
2220 reflections	(Δ/σ)_max_ < 0.001
111 parameters	Δρ_max_ = 0.40 e Å^−^^3^
0 restraints	Δρ_min_ = −0.22 e Å^−^^3^

### Special details

**Table:**

Geometry. All esds (except the esd in the dihedral angle between two l.s. planes) are estimated using the full covariance matrix. The cell esds are taken into account individually in the estimation of esds in distances, angles and torsion angles; correlations between esds in cell parameters are only used when they are defined by crystal symmetry. An approximate (isotropic) treatment of cell esds is used for estimating esds involving l.s. planes.
Refinement. Refinement of *F*^2^ against ALL reflections. The weighted *R*-factor wR and goodness of fit *S* are based on *F*^2^, conventional *R*-factors *R* are based on *F*, with *F* set to zero for negative *F*^2^. The threshold expression of *F*^2^ > 2σ(*F*^2^) is used only for calculating *R*-factors(gt) etc. and is not relevant to the choice of reflections for refinement. *R*-factors based on *F*^2^ are statistically about twice as large as those based on *F*, and *R*- factors based on ALL data will be even larger.

### Fractional atomic coordinates and isotropic or equivalent isotropic displacement parameters (Å^2^)

**Table:**

	*x*	*y*	*z*	*U*_iso_*/*U*_eq_	
Si1	−0.03046 (4)	0.64331 (9)	0.08538 (2)	0.02208 (16)	
H1	0.0590	0.5194	0.1223	0.026*	
C1	−0.00698 (14)	0.8349 (3)	0.03690 (8)	0.0207 (4)	
C2	−0.09110 (15)	0.8753 (3)	−0.02358 (8)	0.0220 (4)	
H2	−0.1545	0.7887	−0.0403	0.026*	
C3	0.08631 (14)	0.9637 (3)	0.06040 (8)	0.0202 (4)	
C4	0.18299 (14)	0.9316 (3)	0.12353 (8)	0.0208 (4)	
C5	0.21644 (16)	1.0962 (3)	0.16753 (9)	0.0276 (4)	
H5	0.1762	1.2298	0.1578	0.033*	
C6	0.30788 (17)	1.0685 (4)	0.22563 (9)	0.0314 (5)	
H6	0.3292	1.1819	0.2555	0.038*	
C7	0.36813 (15)	0.8762 (4)	0.24023 (8)	0.0285 (4)	
H7	0.4314	0.8582	0.2798	0.034*	
C8	0.33562 (16)	0.7103 (4)	0.19677 (9)	0.0310 (5)	
H8	0.3763	0.5773	0.2067	0.037*	
C9	0.24395 (16)	0.7377 (3)	0.13896 (8)	0.0261 (4)	
H9	0.2223	0.6231	0.1094	0.031*	
C10	−0.0605 (2)	0.8174 (4)	0.13337 (10)	0.0375 (5)	
H10A	−0.1249	0.9065	0.1068	0.056*	
H10B	0.0002	0.9164	0.1592	0.056*	
H10C	−0.0726	0.7209	0.1593	0.056*	
C11	−0.14236 (19)	0.4490 (4)	0.03752 (10)	0.0377 (5)	
H11A	−0.1565	0.3595	0.0637	0.057*	
H11B	−0.1230	0.3509	0.0151	0.057*	
H11C	−0.2068	0.5338	0.0084	0.057*	

### Atomic displacement parameters (Å^2^)

**Table:**

	*U*^11^	*U*^22^	*U*^33^	*U*^12^	*U*^13^	*U*^23^
Si1	0.0233 (3)	0.0233 (3)	0.0197 (3)	0.0028 (2)	0.0118 (2)	0.0051 (2)
C1	0.0221 (9)	0.0217 (9)	0.0194 (8)	0.0021 (7)	0.0120 (7)	0.0018 (7)
C2	0.0213 (9)	0.0241 (9)	0.0199 (8)	−0.0012 (7)	0.0110 (7)	−0.0005 (7)
C3	0.0208 (9)	0.0228 (9)	0.0162 (8)	0.0024 (7)	0.0096 (7)	0.0009 (7)
C4	0.0190 (8)	0.0251 (9)	0.0170 (8)	0.0005 (7)	0.0090 (7)	0.0016 (7)
C5	0.0268 (10)	0.0246 (10)	0.0242 (9)	0.0041 (8)	0.0094 (8)	−0.0009 (8)
C6	0.0315 (11)	0.0333 (11)	0.0202 (9)	0.0000 (9)	0.0082 (8)	−0.0051 (8)
C7	0.0202 (9)	0.0406 (12)	0.0174 (8)	0.0028 (8)	0.0056 (7)	0.0015 (8)
C8	0.0259 (10)	0.0365 (11)	0.0242 (9)	0.0115 (9)	0.0096 (8)	0.0038 (9)
C9	0.0276 (10)	0.0274 (10)	0.0199 (9)	0.0051 (8)	0.0108 (8)	−0.0020 (8)
C10	0.0482 (13)	0.0381 (12)	0.0341 (11)	0.0045 (10)	0.0275 (11)	0.0008 (10)
C11	0.0454 (13)	0.0356 (12)	0.0367 (12)	−0.0103 (10)	0.0254 (11)	−0.0035 (10)

### Geometric parameters (Å, °)

**Table:**

Si1—C11	1.851 (2)	C6—C7	1.383 (3)
Si1—C10	1.866 (2)	C6—H6	0.9500
Si1—C1	1.8812 (19)	C7—C8	1.385 (3)
Si1—H1	1.3623	C7—H7	0.9500
C1—C2	1.400 (2)	C8—C9	1.384 (3)
C1—C3	1.405 (3)	C8—H8	0.9500
C2—C3^i^	1.394 (3)	C9—H9	0.9500
C2—H2	0.9500	C10—H10A	0.9800
C3—C2^i^	1.394 (3)	C10—H10B	0.9800
C3—C4	1.494 (2)	C10—H10C	0.9800
C4—C5	1.386 (3)	C11—H11A	0.9800
C4—C9	1.395 (3)	C11—H11B	0.9800
C5—C6	1.387 (3)	C11—H11C	0.9800
C5—H5	0.9500		
			
C11—Si1—C10	110.47 (11)	C5—C6—H6	119.9
C11—Si1—C1	111.24 (9)	C6—C7—C8	119.55 (17)
C10—Si1—C1	108.04 (10)	C6—C7—H7	120.2
C11—Si1—H1	107.7	C8—C7—H7	120.2
C10—Si1—H1	109.4	C9—C8—C7	120.16 (19)
C1—Si1—H1	110.0	C9—C8—H8	119.9
C2—C1—C3	117.29 (16)	C7—C8—H8	119.9
C2—C1—Si1	118.99 (14)	C8—C9—C4	120.83 (18)
C3—C1—Si1	123.17 (13)	C8—C9—H9	119.6
C3^i^—C2—C1	123.17 (17)	C4—C9—H9	119.6
C3^i^—C2—H2	118.4	Si1—C10—H10A	109.5
C1—C2—H2	118.4	Si1—C10—H10B	109.5
C2^i^—C3—C1	119.54 (16)	H10A—C10—H10B	109.5
C2^i^—C3—C4	117.97 (16)	Si1—C10—H10C	109.5
C1—C3—C4	122.47 (16)	H10A—C10—H10C	109.5
C5—C4—C9	118.37 (16)	H10B—C10—H10C	109.5
C5—C4—C3	121.00 (17)	Si1—C11—H11A	109.5
C9—C4—C3	120.59 (17)	Si1—C11—H11B	109.5
C4—C5—C6	120.91 (18)	H11A—C11—H11B	109.5
C4—C5—H5	119.5	Si1—C11—H11C	109.5
C6—C5—H5	119.5	H11A—C11—H11C	109.5
C7—C6—C5	120.18 (19)	H11B—C11—H11C	109.5
C7—C6—H6	119.9		
			
C11—Si1—C1—C2	23.44 (19)	C1—C3—C4—C5	−114.5 (2)
C10—Si1—C1—C2	−97.97 (17)	C2^i^—C3—C4—C9	−110.8 (2)
C11—Si1—C1—C3	−165.30 (16)	C1—C3—C4—C9	67.7 (2)
C10—Si1—C1—C3	73.29 (18)	C9—C4—C5—C6	−0.4 (3)
C3—C1—C2—C3^i^	−0.6 (3)	C3—C4—C5—C6	−178.25 (18)
Si1—C1—C2—C3^i^	171.16 (14)	C4—C5—C6—C7	0.9 (3)
C2—C1—C3—C2^i^	0.6 (3)	C5—C6—C7—C8	−1.0 (3)
Si1—C1—C3—C2^i^	−170.81 (14)	C6—C7—C8—C9	0.6 (3)
C2—C1—C3—C4	−177.92 (17)	C7—C8—C9—C4	0.0 (3)
Si1—C1—C3—C4	10.7 (3)	C5—C4—C9—C8	0.0 (3)
C2^i^—C3—C4—C5	67.0 (2)	C3—C4—C9—C8	177.82 (19)

Symmetry codes: (i) −*x*, −*y*+2, −*z*.
